# The Influence of Weather Conditions on the Diurnal Variation in Range of Motion in Older Adults with Knee Osteoarthritis

**DOI:** 10.3390/jcm13010254

**Published:** 2024-01-01

**Authors:** Elena Ioana Iconaru, Emilian Tarcau, Constantin Ciucurel

**Affiliations:** 1 Department of Medical Assistance and Physical Therapy, University Center of Pitesti, National University for Science and Technology Politehnica Bucuresti, 110040 Pitesti, Romania; elena_ioana.iconaru@upb.ro (E.I.I.); constantin.ciucurel@upb.ro (C.C.); 2 Department of Physical Education, Sport and Physical Therapy, University of Oradea, 410087 Oradea, Romania

**Keywords:** meteorological influences, chronobiological patterns, gonarthrosis, joint stiffness, joint pain, goniometry, aging

## Abstract

(1) Background: This study investigated the relationship between weather conditions, diurnal patterns, and total knee range of motion (ROM), as well as the severity of symptoms (pain and stiffness) in older adults with knee osteoarthritis. (2) Methods: An exploratory longitudinal study was conducted on 28 older adults with knee osteoarthritis (mean age 71.86 ± 4.49 years; 46.4% men, 53.6% women). We used as assessment tools the Visual Analog Scales (VAS) for self-reported local knee pain and stiffness, and goniometry for ROM. Measurements were taken twice, six months apart, in winter and summer, in the morning and evening of each selected day. Recorded weather factors comprised temperature, relative humidity, barometric pressure, and maximum wind speed. (3) Results: The study revealed significant effects of season and time of day on pain and stiffness, respectively (*p* < 0.001). Additionally, a significant interaction between season and time influenced total knee ROM (*p* < 0.001). Moreover, there was a statistically significant relationship between time and total knee ROM (*p* < 0.001). (4) Conclusions: This research underscores the complex link between seasonal fluctuations and daily variations in some symptomatic and functional aspects of knee osteoarthritis in older adults.

## 1. Introduction

In recent decades, there has been a significant increase in scientific interest in the impact of weather conditions on individuals’ and communities’ health. Many comprehensive reviews have indicated a connection between climate change and declining human health [1]. Also, the aging population and the rising incidence of degenerative musculoskeletal disorders, such as knee osteoarthritis, have been the subject of numerous studies [2].

Knee osteoarthritis, a widespread type of arthritis, exerts a significant clinical impact on the lower limb, given the biomechanical significance of this joint for locomotion and everyday activities. The prevalence of knee osteoarthritis increases with age [3], reflecting cumulative exposure to risk factors and age-related changes in the joints. Identified risk factors in this context include age, female gender, obesity, previous knee injury, knee malalignment, and weakness in knee extensor muscles [4]. The condition also appears in younger populations due to factors such as obesity, sports, genetics, occupation, prior injuries, or socio-economic status [5]. Regardless of causality, cartilage damage serves as a significant diagnostic marker for disease staging [6].

The disease itself leads to chronic pain, morning joint stiffness, crepitus, edema, and marked functional impairment [7]. Slowing knee osteoarthritis progression in older adults is currently of major interest to health policymakers due to its significant public health impact, given our aging population and increased risk factors like obesity [8].

Currently, the disease is relatively easy to diagnose and stage from a clinical perspective, but especially through paraclinical means [9]. Our study, while focusing on classic diagnostic tools, acknowledges the emergence of novel alternative approaches, such as vibroacoustics [10]. This method shows promise in detecting subtle changes in joint structures and function at an early stage [11]. Vibroarthrography, proposed as a harmless, noninvasive, and accurate tool for evaluating cartilage, offers various optimized examination protocols for achieving superior diagnostic outcomes [12]. However, there are still gaps in our understanding regarding the progression of the disease and its correlation with particular risk factors. Identifying knee osteoarthritis early in patients and addressing associated risk factors are very important because they allow for the initiation of appropriate treatment in the first stages of the disease [13]. Practically, early recognition enables the implementation of interventions and lifestyle modifications that can slow down the progression of the condition, thereby reducing the severity of symptoms and improving patients’ quality of life by reducing its impact on the patient’s functionality.

Although extensive knowledge about this chronic disease exists, there are still numerous uncertainties surrounding weather sensitivity, the impact of circadian rhythms on the patient’s clinical functional status, and the overall clinical progression concerning both endogenous and exogenous variables. In general, the relationship between degenerative diseases and environmental conditions, as well as the diurnal patterns of certain symptoms, is an intriguing topic. This interest arises from data reported by clinicians based on empirical experience; unfortunately, these pieces of information do not always have rigorous scientific support. Even in classical medical textbooks, we find references of this kind. Some assumptions relate to the intricate connections between environmental factors and the progression of such diseases, presupposing the existence of a complex interplay between biology and the surrounding world.

For example, Hippocrates observed and documented the influence of meteorological factors on health in his manuscript “Air, Water, and Places”, suggesting that sudden weather changes can impact human well-being [14]. These principles, as discussed in ancient medical texts, have significantly influenced contemporary healthcare practices. They highlight the relationship between a hygienic environment, public health, and overall human well-being, emphasizing the responsibility of society to ensure a healthy living environment [15].

Regarding circadian biological rhythms and human health, the literature is abundant with studies and research. Circadian rhythms are inherent patterns that repeat daily, sustaining themselves through synchronization and oscillation, governing various molecular, physiological, and behavioral processes, and implicating pathological conditions [16]. Indeed, research has indicated that strengthening the intensity of circadian rhythms can profoundly enhance one’s health and overall sense of well-being [17]. Furthermore, these biological rhythms are continually influenced by environmental conditions, adapting organisms’ behaviors and physiology to changes in light–dark cycles, day lengths, and weather patterns [18].

When discussing a musculoskeletal degenerative disease like knee osteoarthritis, the question arises: to what extent can we identify meteorological factors and diurnal rhythms that could explain fluctuations in symptoms and functionality in individuals with this condition? Therefore, we deemed it opportune to conduct a longitudinal study of the influence of environmental climatic conditions and diurnal variations on the functional status of older adults with knee osteoarthritis. More precisely, this study aimed to explore the relationship between factors like meteorological conditions (in terms of air temperature, humidity, barometric pressure, and maximum wind speed), chronobiological patterns (referring to daily rhythms), and the range of motion (ROM), along with the severity of symptoms experienced by older individuals with knee osteoarthritis.

## 2. Materials and Methods

### 2.1. Participants and Study Design

This study employed an exploratory longitudinal design involving a sample of 28 older adults diagnosed with knee osteoarthritis (mean age 71.86 ± 4.49 years; age range 65–79 years; 13 men and 15 women). Participants were assessed at their place of residence. The research comprised two consecutive assessments conducted at a 6-month interval: the first between January 15–17 and the second between July 15–17. Both assessment periods coincided with the midpoint of winter and summer in Romania. Despite its size, the sample was carefully selected to align with previous studies and ensure statistical robustness [19,20]. Our statistical methodologies further support the reliability of our findings within the confines of the sample size.

As for the clinical characteristics of the participants, 42.86% exhibited bilateral gonarthrosis. All cases of gonarthrosis were categorized within stages I–III according to The Kellgren–Lawrence Classification of Osteoarthritis [21,22], and the stage did not change between the two assessments. The decision to focus on individuals in these earlier clinical stages was based on the rationale that they often manifest milder symptoms and functional limitations compared to those in advanced stages. We opted to exclude patients with advanced knee osteoarthritis due to the challenges they posed in assessing joint mobility and interpreting disease progression within the context of our experimental design.

Inclusion and exclusion criteria: in addition to obtaining written informed consent, participants were required to have permanent residence in the last year within the geographical location where environmental parameters were determined. Further, participants should not have received knee osteoarthritis treatment during the week preceding each evaluation. Exclusion criteria encompassed any reported acute illness within the last two weeks, participants with a history of knee surgery in the past six months, individuals with diagnosed severe cardiovascular or respiratory conditions, subjects undergoing active cancer treatment or diagnosed with malignancies, participants with a history of chronic inflammatory diseases affecting joint health, and participants with significant cognitive impairment affecting their ability to comprehend study procedures and provide informed consent.

### 2.2. Data Acquisition

For the study, we chose to collect data enabling a comprehensive analysis of the relationship between meteorological conditions, individual categorical factors, and clinical parameters. Concerning environmental conditions, we recorded the mean external temperature (in degrees Celsius), relative humidity (in percentage), barometric pressure (in hPa), and maximum wind speed (in m/s) over three consecutive days during the previously mentioned periods. The location where the recordings were taken is situated at an average altitude of 250 m, in an urban area experiencing a temperate oceanic climate (Köppen Classification: Cfb) [23]. The measurements were taken in the morning at 8 a.m. and in the evening at 6 p.m. using the Bresser WiFi HD TFT Professional Weather Station with a 7-in-1 Sensor. The manufacturer of the station specifies a good measurement accuracy for the monitored variables [24]. Such stations, with Wi-Fi data transmission capabilities, are user-friendly, reliable for quick field assessments [25], and can be successfully employed in climatological scientific research [26]. The recorded values were averaged per day and then over the three consecutive days.

The patients were initially evaluated clinically and paraclinically before inclusion in the study sample to ensure eligibility. Subsequently, clinical assessments of the subjects were conducted at two time points on the same day, for each considered calendar date, in an enclosed space. Thus, the first evaluation of the day took place between 8–9 a.m., after the patient woke up, and attended to their physiological needs, but before breakfast. The second evaluation of the day was conducted between 5–6 p.m., without any prior conditions. Subjects were allowed to engage in their usual daily activities, with occasional outings in open spaces, but without excessive physical exertion on that specific day. At the time of evaluations, the ambient temperature was comfortable, around 22–23 °C, and the relative room humidity ranged between 40 and 60%.

Clinical evaluations were conducted using two Visual Analog Scales (VAS) to assess local knee pain and stiffness. On a scale from 0 to 10, each patient first rated pain on movement and then the stiffness in their knees individually. In the case of patients with bilateral gonarthrosis, they assessed the knee most affected. VAS is a straightforward and efficient tool for patients to rate the intensity of their pain or joint stiffness. It helps professionals understand pain and stiffness levels, allowing them to track changes over time and aiding in proper treatment and communication between patients and healthcare providers [27,28]. VAS has strong reliability references, showing minimal errors in assessing knee pain related to osteoarthritis [29].

Following these subjective evaluations, the ROM of the affected or most affected knee joint was determined for each patient. This process involved using a goniometer, with the patient in a supine position, extending or flexing the knee while keeping the contralateral knee bent [30]. Correct goniometer placement, aligned with anatomical landmarks, ensured precise measurements. The stationary arm of the goniometer was positioned parallel to the femur bone, and the moving arm was parallel to the fibula. Extension and flexion angles were measured, and the total knee ROM was calculated by adding the flexion angle to the extension angle [31,32]. Goniometry, used to assess knee mobility, is considered a clinical method with high accuracy [33].

For reporting goniometry data, we considered the conventional understanding of normal knee ROM, stated by most authors, to be 0° of extension and 135° of flexion [31]. Studies also confirm that during active flexion, the knee can reach 120°–140° with the hip flexed, while it can reach up to 160° passively [34]. However, some authors suggest that most people exhibit hyperextension (about 5° in males and 6° in females), challenging the traditional concept of knee joint mobility [35]. In our case, we recorded active movements, not passive ones, as these are common in activities of daily living [36].

For each subject, we followed standard procedures to measure weight (W, in kilograms) and height (H, in meters). Using these measurements, we calculated the body mass index (BMI, in kg/m^2^) to determine their weight status in accordance with the NIH/WHO guidelines [37].

### 2.3. Statistical Analysis of Data

This study provided data resulting from environmental measurements (temperature, relative humidity, barometric pressure, and maximum wind speed) and data resulting from measurements performed on the subjects (age, sex, weight, height, BMI, VAS score for pain, VAS score for stiffness, and total ROM for the knee). The human parameters were determined for each subject twice, under specific mid-winter and mid-summer environmental conditions. For each season, two measurements were taken on the same day, in the morning and in the evening, to assess the diurnal variations in the weather parameters. The dataset was processed using the IBM SPSS 26.0 software (IBM Corp., Armonk, NY, USA) [38]. Descriptive statistical techniques, including computations of mean values, standard deviations, and frequency distributions, were applied. Additionally, we assessed the normality of data distribution using the Shapiro–Wilk test. Furthermore, inferential statistical approaches, specifically two-way repeated measures Analysis of Variance (ANOVA), were employed to extract nuanced insights from the data, thereby enhancing the depth of the study’s findings. The method was applied separately for each of the three variables: VAS pain score, VAS stiffness score, and total ROM for the knee, in relation to two factors: season (winter or summer) and time of day (morning or evening). Through this analysis, we thoroughly examined how “seasonal conditions” (the first factor) and “time of day” (the second factor) interacted and influenced each dependent variable. We also calculated the effect size, specifically Partial Eta-Squared (η^2), to assess the magnitude of the observed effects in the study and the Observed Power.

## 3. Results

For the analysis and presentation of the results, it is essential to specify the weather conditions during the two evaluation periods in winter, from 15–17 January, and summer, from 15–17 July (Table 1).

Overall, we can observe that during winter, the weather conditions were characterized by cold temperatures (mean day of 5 °C), high relative humidity (around 88%), some fluctuations in barometric pressure, and low maximum wind speed. These conditions are typical of winter, creating a chilly and often damp environment. In contrast, the summer period exhibited significantly warmer temperatures (mean day of 28.2 °C) and much lower relative humidity (approximately 53%). The barometric pressure remained stable, also exhibiting a relatively low peak wind velocity. This combination of warm temperatures, low humidity, and dry winds is characteristic of summer weather patterns. Comparatively, the winter season presented a colder, moister atmosphere, while summer was marked by warmth and dryness.

The data presented in Table 1 align with the typical climatic conditions of the research location [39]. This ensures contextual harmony in the data and reduces the likelihood of outlier effects during analysis due to distinct weather conditions. Given the ongoing debate on how seasonal effects impact joint conditions [40], we aim to highlight the specific influences of weather conditions on knee osteoarthritis by separating summer and winter seasons.

In this study, a sample of 28 older adults was analyzed, with 46.4% of the participants being men and 53.6% being women. Their anthropometric profiles are synthesized as descriptive statistics and presented in Table 2.

Regarding BMI values, 39.3% of the subjects were classified as having normal BMI, 46.4% were overweight, 10.7% had class 1 obesity, and 3.6% were underweight. It is important to note that the statistical analysis did not focus on the nutritional status of the subjects or sex-specific characteristics, given the relatively small size of the sample.

In Table 3 below, the mean values and standard deviations of the variables recorded for the study samples are synthetically presented for the two seasons (winter and summer) and the two time points (morning and evening). These data were subjected to statistical analysis using a two-way ANOVA with repeated measures, and their interpretation was conducted considering this type of analysis. Before conducting the ANOVA, the Shapiro–Wilk test was used to assess the normality of the data. The results indicated an approximately normal distribution, which was satisfactory to run an ANOVA based on the required assumption.

The purpose of the two-way ANOVA with repeated measures analysis was to assess the impact of seasonal variations and different times of the day on VAS pain scores, VAS stiffness scores, and total ROM for the knee. This method allows us to discern not only the main effects of each factor but also their interaction effect [41], providing a comprehensive understanding of how these variables are influenced by both seasonal changes and the specific moments within a day. The aim of the two-way ANOVA with repeated measures analysis was to discern significant differences and patterns in the data, in order to clarify the relationship between the variables under investigation. The results of this analysis are summarized in Table 4.

**Figure 1:**
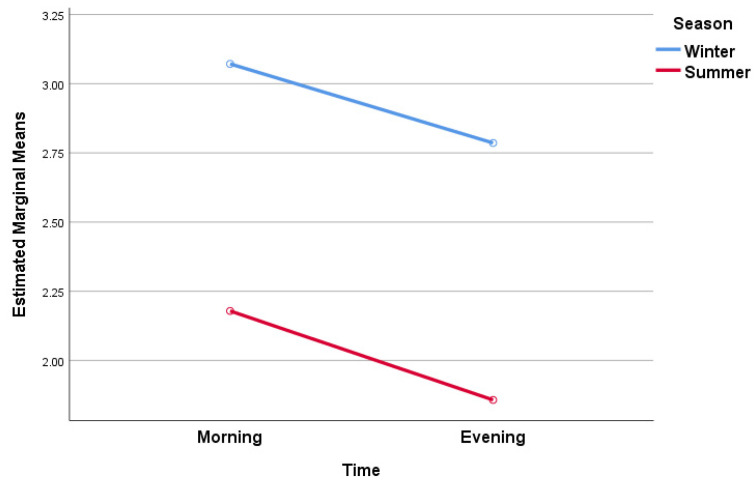
Estimated marginal means of VAS pain score.

Initially, we conducted a two-way ANOVA with repeated measures analysis to assess the influence of both seasonal variations (winter or summer) and diurnal changes (morning or evening) on the self-perception of pain intensity. Our aim was to determine whether the season could affect the diurnal variation in pain, as measured using the VAS pain scale, in older adults with knee osteoarthritis. The two within-subject factors were the season (winter or summer) and the time of day (morning or evening), with the dependent variable being the VAS pain score. In terms of variables, our focus was to understand if there was an interaction between the season and time of day, taking into account the VAS pain score (Figure 1).

We can observe that the main effects of the season are statistically significant at *p* < 0.001, with Partial Eta Squares (0.432) indicating large effect sizes (values higher than 0.40) [42]. Additionally, in this case, the Observed Power (0.992) was greater than 80% and therefore, we can conclude that the sample size was adequate. However, the main effects of time and the season * time interaction were not statistically significant.

In summary, the season significantly impacts VAS pain scores, explaining a significant part of the variation. However, time and the interaction between season and time did not significantly impact VAS pain scores in this study. As an explanation, the study might not have enough statistical power to detect minor effects related to time and the season * time interaction. In other words, variations in self-reported pain in patients with knee osteoarthritis due to seasons are consistent and independent of the specific time of day when they are assessed. Since the season factor is the sole significant influence of VAS pain scores in our analysis, we can conclude that patients experience pain differently depending on the weather conditions, regardless of the specific time of day. Additionally, patients report higher levels of pain during the cold season compared to the warm season.

**Figure 2:**
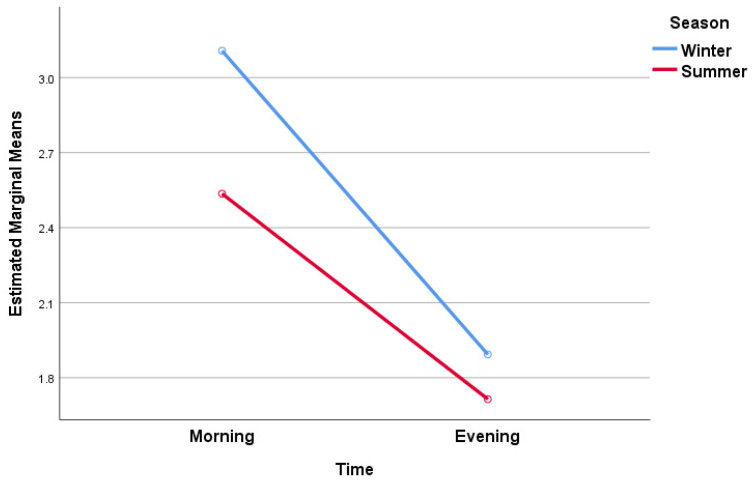
Estimated marginal means of VAS stiffness scores.

Following the same procedure, the subsequent ANOVA analysis investigated the influence of seasonal variations (winter or summer) and diurnal changes (morning or evening) on the self-perception of stiffness, as indicated by VAS scores (Figure 2). In this case, the results were statistically significant only for the main effects related to time (*p* < 0.001), displaying large effect sizes based on Partial Eta Squares (0.768), and a very good Observed Power (1). On the other hand, the main effects of season and the interaction between season and time were not statistically significant.

Therefore, the main conclusion is that the differences observed in VAS scores for stiffness are more influenced by diurnal variability than by seasonal changes or the interaction between season and time of day in this study. Overall, knee joint stiffness in older adults with knee osteoarthritis is higher in the morning compared to the evening, regardless of the season.

**Figure 3:**
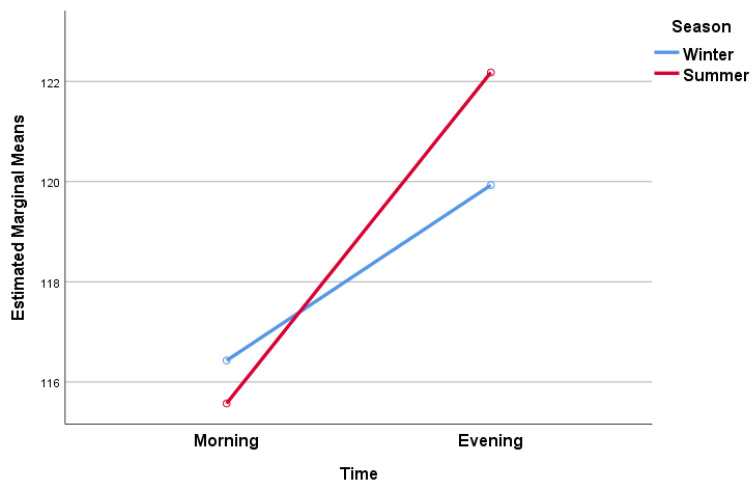
Estimated marginal means of total ROM for the knee.

The last ANOVA analysis focused on the relationship between seasonal variations (winter or summer) and diurnal changes (morning or evening) regarding the total ROM for the knee (Figure 3). This time, we investigated an objective parameter resulting from goniometric measurements, following the experimental design. When presenting the results, it is necessary to interpret the mean values of ROM across the four evaluations in relation to the normality standard of 135° [31]. Consequently, we observed a certain limitation of knee mobility across the entire sample due to underlying pathological changes of osteoarthritis. Furthermore, the ANOVA analysis revealed differences between the analyzed factors.

The results demonstrated statistically significant relationships (*p* < 0.001) between time and the interaction between season and time with total ROM for the knee in older adults with knee osteoarthritis. However, the relationship between season and total ROM for the knee alone was not statistically significant. Specifically, during the winter season, the mean total ROM for the knee in the morning was 116.43° and increased slightly to 119.93° in the evening. In the summer season, the mean ROM was 115.57° in the morning and increased significantly to 122.18° in the evening.

These findings reveal subtle differences in knee joint mobility in the morning and evening during the winter season. However, a more pronounced variation in knee ROM between morning and evening was evident during the summer season, with higher values observed in the evening hours. Although the seasonal factor alone did not exhibit a statistically significant relationship with ROM, the results imply that the influence of season on knee joint mobility becomes more noticeable during the summer, particularly in terms of morning-to-evening variations. The notable effect of the interaction between season and time of day on knee ROM values underscores the impact of season (winter vs. summer) on knee joint mobility fluctuation based on the time of day. While seasonal factors do exert some influence, it was evident that the time of day magnified these disparities, resulting in significant fluctuations in knee mobility.

The results of the last ANOVA analysis also demonstrated a substantial effect size (Partial Eta Squared = 0.745) and excellent statistical power (Observed Power = 1) for the time factor, signifying a robust influence on knee ROM. In contrast, the interaction between season and time exhibited a moderate effect size (Partial Eta Squared = 0.204), with an acceptable level of statistical power (Observed Power = 0.718).

## 4. Discussion

The present study aimed to explore the relationship between the clinical manifestations of knee osteoarthritis in older adults and the combined influence of seasonal variations and diurnal fluctuations. Our intention was to provide evidence contributing to the understanding of the interplay between weather sensitivity and circadian biological rhythms in the context of this degenerative musculoskeletal disorder. This topic is of significant relevance due to the high prevalence of knee osteoarthritis associated with the aging process, along with the multitude of pathophysiological factors that encompass this clinical entity.

In our study, we employed a rigorous experimental design, utilizing a novel approach that involved sequential statistical analysis. We considered two key factors: the seasons (winter and summer) and distinct periods of the day (morning and evening). This holistic perspective proves to be valuable for both clinicians and researchers, offering profound insights into the dynamic nature of the disease and presenting arguments for more targeted interventions and personalized treatment strategies.

Interpreting the results of our study, it becomes evident that both seasonal changes and diurnal variations significantly impact various symptomatic and functional aspects of knee osteoarthritis in older adults. The observed dynamics of VAS pain scores, VAS stiffness scores, and total ROM for the knee argue for the existence of variation patterns in relation to the investigated factors. Our findings align with previous research, indicating the intricate nature of osteoarthritis, where symptomatology is not solely dictated by the severity of joint degeneration but also by external or internal factors such as weather conditions, constitutional factors, and physical activity [43,44].

The significant influence of season on VAS pain scores demonstrated by our research suggests a direct connection between weather conditions and pain perception in older adults with knee osteoarthritis. Our data indicate higher pain levels in winter compared to summer, aligning with studies attributing heightened pain sensitivity to low temperatures and cold weather conditions [45]. Moreover, the lack of significant influence of the time of day on VAS pain scores implies that this sensitivity to seasonal changes persists consistently throughout the day, emphasizing the need for tailored pain management strategies across all hours. Also, the insignificant impact on VAS pain scores of the interaction between season and time in this study might be due to limitations like the small sample size, possibly leading to insufficient statistical power to detect minor effects of these combined factors.

In a recent systematic review and meta-analysis, the link between weather conditions and osteoarthritis pain was underscored. This comprehensive analysis revealed significant correlations between osteoarthritis pain and temperature, barometric pressure, and relative humidity, providing additional support to the body of evidence of the relationship between weather factors and osteoarthritis clinical signs [46]. Our results confirm the aforementioned theory and emphasize the importance of recognizing weather sensitivity in the daily routines of older individuals dealing with osteoarthritis.

Considering that pain from knee osteoarthritis plays a significant role in driving disability, closely associated with the severity of the condition [47], it underscores how pain intensity directly impacts the functional restrictions of these patients. Hence, managing pain in this context, influenced by climatic factors and circadian rhythms, becomes a proactive approach to prevent the onset of disability.

On the other hand, the findings of VAS stiffness scores, influenced by diurnal rhythm, corroborate existing knowledge on this topic [48]. The significant effect of diurnal changes on stiffness, particularly the higher values observed in the morning, underscores the importance of taking patients’ stiffness levels into account when projecting daily routines and habits. Hence, morning stiffness, recognized as a disease marker [49], should be considered when managing these patients, irrespective of the season. This understanding could enhance treatment strategies, potentially incorporating specific exercises or therapies during the morning hours to alleviate stiffness-related discomfort.

The topic of the parallelism between pain and stiffness in knee osteoarthritis is intriguing and warrants further exploration. We have highlighted the weather sensitivity of pain and the diurnal nature of joint stiffness. As a plausible explanation, we can consider the intricate interplay between external environmental factors and internal disease manifestations, such as mechanical stress, inflammation, metabolism, hormonal shifts, and aging, leading to irreversible joint damage [50]. Moreover, weather influences knee osteoarthritis symptoms, emphasizing the complex relationship between external weather conditions and internal disease manifestations, including joint stiffness and pain [44].

Furthermore, the classic phenomenon of joint morning stiffness, associated with the heightened pain extensively documented in numerous studies [51], can be understood through the physiological changes that occur overnight. Reduced joint movement during sleep can lead to the accumulation of synovial fluid, making the joint stiffer in the morning. Additionally, the body’s circadian rhythms, which influence various biological processes, might also play a role, leading to more pronounced joint stiffness during the early hours of the day. The synchronization of neuroendocrine pathways with immune and inflammatory responses, influenced by biological rhythms, could represent an explanation for the phenomena described earlier [52].

Other recent research has highlighted that weather changes affect osteoarthritis symptoms by altering synovial fluid composition and joint lubrication. It appears that increased barometric pressure intensifies discomfort, impacting these patients due to lower pain thresholds. Cold temperatures elevate synovial fluid viscosity and alter periarticular structures’ compliance, making joints stiffer and more sensitive to mechanical stress during activities [53]. These factors aggravate osteoarthritis symptoms, affecting patient mobility and healthcare accessibility [46].

The compelling relationship between pain sensitivity to weather shifts and daily fluctuations in joint stiffness requires extensive investigation. This connection may stem from the complex interplay between external weather elements and internal manifestations of disease. Recent findings highlight the positive association of both daily average humidity and three-day average humidity with joint pain, particularly noting that humidity’s impact on pain was more pronounced under relatively colder weather conditions [54]. Consequently, these discoveries emphasize the necessity for a deeper comprehension of how climatic factors intersect with internal bodily responses across different environmental conditions, influencing the clinical manifestation and progression of the disease.

The final aspect that needs discussing pertains to the total ROM for the knee. Examining this variable in detail enhances our understanding of how functionality in patients with osteoarthritis is influenced by environmental and temporal factors. Interestingly, while season alone did not directly impact knee mobility, the interaction between season and time of day, as well as the time of day as an independent factor, determines intriguing patterns of knee ROM. Notably, there was a significant increase in ROM during summer evenings, suggesting a potential beneficial effect of warmer weather on joint flexibility. This contrasts with the more limited variation observed between morning and evening ROM in winter, indicating more stable but restricted mobility throughout the day in colder months.

The differences in knee ROM in the morning and the evening, especially in summer, might be attributed to temperature fluctuations or other factors. While temperature significantly impacts joint flexibility, with heat being potentially beneficial for muscle and ligament flexibility [55], numerous other elements could contribute to this circadian variation. Daily movement tends to peak during the day, leading to improved joint flexibility and mobility in the evening due to hormonal adaptations induced by movement patterns [56]. For example, the lower cortisol levels in the evening compared to the morning may suggest a potential improvement in skeletal muscle metabolism towards the evening. Studies indicate that hormonal response to exercise varies throughout the day, favoring an anabolic profile later in the day [57]. Consequently, periarticular muscle structures may positively influence joint function, potentially increasing the ROM in the evening.

In the same context, over the course of the day, muscle relaxation could potentially increase due to reduced stress and activity levels, leading to potential enhancement in joint mobility. Although the exact mechanism driving the increased knee ROM remains uncertain, modifications in muscle stiffness have been linked to this alteration [58]. Furthermore, the body’s adjustment to daily activities might impact joint flexibility. Moreover, the body’s hydration status across the day might impact tissue elasticity, consequently influencing the ability to move the joints. Research suggests that in typical individuals, hydration levels exhibit a circadian rhythm [59] and this aspect could also potentially interfere with the previously described mechanisms.

Lastly, variations in inflammation levels, which fluctuate throughout the day, could impact joint function and flexibility. The circadian clock regulates cellular metabolism and orchestrates immune cell activity, influencing the body’s immune response [60]. For instance, in patients with rheumatoid arthritis, joint stiffness follows a diurnal rhythm, peaking in the morning, reflecting the disease’s circadian nature. Inflammatory cytokines, predominantly secreted early in the morning, play a significant role in causing morning stiffness [61]. Therefore, the diurnal fluctuations in ROM in patients with knee osteoarthritis might be partially explained from an immunological perspective. However, these combined factors collectively contribute to the observed changes in knee ROM from morning to evening, especially during the summer months.

We can understand these ROM dynamics in older adults with knee osteoarthritis by referring to similar studies conducted in healthy individuals. Therefore, studies have shown a diurnal cycle in knee joint cartilage thickness, influenced by specific locations experiencing the highest biomechanical forces [62]. Cartilage thickness generally decreases from morning to evening in most parts of the knee, except the patellofemoral groove, which can alter the compression forces occurring in the femoral condyles and tibial plateau. These findings are valuable for understanding joint homeostasis and potentially the onset of abnormal movements, promoting joint degeneration [63].

It is interesting to interpret the current research results in relation to studies conducted on other anatomical segments of the human body, which have sometimes yielded controversial outcomes. For example, a study analyzing the daily flexibility patterns of hamstrings and the lumbar region, considering gender differences, highlights the need for careful consideration and control of diurnal and gender-related variations in flexibility assessments, training, and research [64]. However, other authors have not identified normal human circadian flexibility in the lumbar spine, although some changes in sensorimotor trunk functions have been reported in individuals with prolonged sitting in the context of office work [65].

Certainly, when examining the knee joint in older adults with osteoarthritis, it becomes clear that understanding the seasonal and daily fluctuations in ROM, which are closely related to clinical symptoms like pain and stiffness, is more cohesive and well-supported. The results of our study provide strong evidence for this comprehension, highlighting the intricate interconnection between weather conditions, the time of day, and joint characteristics in the context of knee osteoarthritis.

Our results have several practical implications. In terms of pain and treatment management, recognizing the diurnal rhythm in knee ROM of older adults with gonarthrosis and its seasonal influences could assist physicians and physical therapists in creating more effective treatment plans. They could customize physical therapy, exercises, and medications to maximize ROM during peak natural periods, potentially improving pain management and knee joint function. Regarding patient education, individuals with knee osteoarthritis could benefit from understanding these diurnal and seasonal ROM patterns to better handle daily activities. They might choose activities requiring extensive knee movements during peak ROM periods, potentially reducing discomfort and pain. For daily activity planning, people with knee osteoarthritis could organize their daily activities based on these ROM rhythms. Activities demanding intense knee movements could be scheduled during peak ROM periods to minimize discomfort or pain, thereby contributing to the psychological well-being of the patient [66].

As a result of our research, our findings provide a perspective for in-depth exploration of the mechanisms underlying diurnal and seasonal ROM rhythms and clinical patterns in osteoarthritic knees. Such research has the potential to optimize osteoarthritis management, offering personalized therapies. For example, these patterns can be explored at a molecular and biomechanical level, which can enhance our understanding of the disease and potentially redefine targeted therapies. Analyzing ROM fluctuations and variations in symptoms and biomarkers, as well as their connection to weather status and patient outcomes, could provide new evidence supporting our epidemiological knowledge of this condition.

It is important to acknowledge the limitations of our study, such as the relatively small sample size and the focus on specific early stages of knee osteoarthritis. However, efforts were directed to statistical analysis to mitigate the impact of this limitation on our study’s findings. This included employing robust methodologies to derive meaningful insights, despite the constrained sample size. Additionally, as with other limitations, assessments of pain and stiffness were conducted using tools reliant on respondents’ subjectivity. Also, the knee ROM measurements were taken by different physiotherapists, introducing challenges related to intervariability in the tests. Future research endeavors could explore larger and more diverse cohorts, encompassing different stages of the condition and considering additional variables like physical activity levels and psychosocial factors. Furthermore, conducting long-term longitudinal studies that monitor patients over extended durations could offer valuable insights into the lasting effects of seasonal and diurnal variations on disease progression and, consequently, on the overall quality of life.

## 5. Conclusions

Knee osteoarthritis in the context of aging remains highly debated. Beyond the traditional knowledge about its etiopathogenesis and dynamics, studies shedding light on factors such as seasonal influences and diurnal rhythms hold great potential for providing clinical evidence for the management of the disease. Our research emphasizes the complex interplay between weather patterns, time of day, and the symptoms and functional aspects of knee osteoarthritis. Specifically, the main effects of the season (winter or summer) on self-reported pain using the VAS scale are statistically significant (*p* < 0.001). Regarding VAS stiffness scores, the results indicate the main significant effects of time (morning or evening) (*p* < 0.001). Furthermore, there is a statistically significant relationship (*p* < 0.001) between time and the interaction between season and time with the total ROM for the knee in older adults with knee osteoarthritis.

Our study reveals a clear influence of both seasonal fluctuations and daily variations on clinical features of knee osteoarthritis in older adults. Recognizing these intricate relationships could enhance healthcare strategies, enabling more precise interventions. Further research has the potential to refine our understanding of knee osteoarthritis mechanisms, providing evidence for personalized care. Identifying seasonal and diurnal patterns in clinical features could significantly impact disease management, ultimately enhancing the quality of life of affected patients, especially older adults.

## Figures and Tables

**Table 1:** Summary of weather conditions during the two periods of evaluation of participants.

Variable	Mean Temperature (°C)	Mean Relative Humidity (%)	Mean Barometric Pressure (hPa)	Maximum Wind Speed (m/s)
Winter				
15 January	4.7	88	1010	15
16 January	4.1	87	1000	9
17 January	6.2	89	992	15
Mean	5.0	88.0	1000.7	13.0
SD	1.1	1.0	9.0	3.5
Summer				
15 July	26.7	58	1007	26
16 July	28.4	53	1006	15
17 July	29.5	48	1005	17
Mean	28.2	53.0	1006.0	19.3
SD	1.2	4.1	0.8	4.8

Note: SD, standard deviation.

**Table 2:** Anthropometric profiles of the study samples (*n* = 28).

Variable	Age (Years)	W (kg)	H (cm)	BMI (kg/m^2^)
Mean	71.86	69.50	165.86	25.30
SD	4.49	11.64	9.70	4.08
Min	65	45	151	18.11
Max	79	93	187	34.93

Note: W, weight; H, height; BMI, body mass index; SD, standard deviation; Min, minimum value; Max, maximum value; *n*, group size.

**Table 3:** Mean values of the measured variables ± SD in the study samples (*n* = 28).

Variable	VAS Pain Score	VAS Stiffness Score	Total ROM for Knee (°)
Winter			
Morning	3.07 ± 1.15	3.11 ± 0.92	116.43 ± 12.21
Evening	2.79 ± 1.32	1.89 ± 0.92	119.93 ± 11.95
Summer			
Morning	2.18 ± 0.72	2.54 ± 0.75	115.57 ± 11.26
Evening	1.86 ± 0.97	1.71 ± 0.76	122.18 ± 11.92

Note: SD, standard deviation; VAS, visual analogic scale; ROM, range of motion; *n*, group size.

**Table 4:** Results of tests of within-subject effects.

Parameter	Effect	Type III Sum of Squares	df	Mean Square	F	*p*-Value	Partial Eta Squared	Observed Power
VAS pain score	Season	23.22	1	23.22	20.54	0.001	0.432	0.992
	Time	2.58	1	2.58	2.66	0.114	0.090	0.350
	Season * Time	0.009	1	0.009	0.012	0.913	0	0.051
VAS stiffness score	Season	3.94	1	3.94	4.119	0.052	0.132	0.499
	Time	29.01	1	29.01	89.605	0.001	0.768	1
	Season * Time	1.08	1	1.08	2.302	0.141	0.079	0.31
Total ROM for the knee	Season	13.58	1	13.58	0.27	0.609	0.10	0.079
	Time	715.08	1	715.08	78.75	0.001	0.745	1
	Season * Time	67.58	1	67.58	6.92	0.014	0.204	0.718

Note: df, the degrees of freedom in the source.

## Data Availability

The data are available on request from the corresponding author. All data relevant to the study are included in the article.
